# Soluble uric acid increases PDZK1 and ABCG2 expression in human intestinal cell lines via the TLR4-NLRP3 inflammasome and PI3K/Akt signaling pathway

**DOI:** 10.1186/s13075-018-1512-4

**Published:** 2018-02-07

**Authors:** Mo Chen, Xiaoyong Lu, Ci Lu, Ning Shen, Yujie Jiang, Menglu Chen, Huaxiang Wu

**Affiliations:** 1 Department of Rheumatology, Second Affiliated Hospital, School of Medicine, Zhejiang University, 310009 Hangzhou, China; 2 Department of Nephrology, Hangzhou Hospital of Traditional Chinese Medicine, 310007 Hangzhou, China; 3 Department of Rheumatology, Sir Run Run Shaw Hospital, School of Medicine, Zhejiang University, 310009 Hangzhou, China

**Keywords:** Hyperuricemia, ABCG2, PDZK1, Intestine

## Abstract

**Background:**

In addition to the kidney, the intestine is one of the most important organs involved in uric acid excretion. However, the mechanism of urate excretion in the intestine remains unclear. Therefore, the relationship between soluble uric acid and the gut excretion in human intestinal cells was explored. The relevant signaling molecules were then also examined.

**Methods:**

HT-29 and Caco-2 cell lines were stimulated with soluble uric acid. Western blotting and qRT-PCR were used to measure protein and mRNA levels. Subcellular fractionation methods and immunofluorescence were used to quantify the proteins in different subcellular compartments. Flow cytometry experiments examined the function of ATP-binding cassette transporter, subfamily G, member 2 (ABCG2). Small interfering RNA transfection was used to assess the interaction between ABCG2 and PDZ domain-containing 1 (PDZK1).

**Results:**

Soluble uric acid increased the expression of PDZK1 and ABCG2. The stimulation of soluble uric acid also facilitated the translocation of ABCG2 from the intracellular compartment to the plasma membrane and increased its transport activity. Moreover, the upregulation of PDZK1 and ABCG2 by soluble uric acid was partially decreased by either TLR4-NLRP3 inflammasome inhibitors or PI3K/Akt signaling inhibitors. Furthermore, PDZK1 knockdown significantly inhibited the expression and transport activity of ABCG2 regardless of the activation by soluble uric acid, demonstrating a pivotal role for PDZK1 in the regulation of ABCG2.

**Conclusions:**

These findings suggest that urate upregulates the expression of PDZK1 and ABCG2 for excretion in intestinal cells via activating the TLR4-NLRP3 inflammasome and PI3K/Akt signaling pathway.

**Electronic supplementary material:**

The online version of this article (10.1186/s13075-018-1512-4) contains supplementary material, which is available to authorized users.

## Background

Pathological hyperuricemia is defined as a serum urate concentration (408 μmol/L) above which monosodium urate (MSU) crystals form at physiological pH and temperature [1]. Persistent hyperuricemia is widely considered the primary risk factor in several gout-associated diseases, such as gouty arthritis, gouty tophi, and renal damage [2]. An increasing trend in the prevalence of gout and hyperuricemia has been revealed by epidemiological investigations in several western countries [3, 4]. In Italy, the prevalence of hyperuricemia increased from 85.4 per 1000 inhabitants in 2005 to 119.3 per 1000 inhabitants in 2009 [5]. Therefore, it is crucial to explore the pathophysiological process of hyperuricemia and its effect on target organs.

Underexcretion is the main reason for hyperuricemia in patients with gout [6]. Renal excretion accounts for approximately two-thirds of urate excretion, whereas gut excretion accounts for the rest [2, 7]. This process is regulated by a variety of apically and basolaterally expressed reabsorptive and secretory transporters, some of which could act as urate-lowering agents [7]. In the gut, the secretory transporter ATP-binding cassette transporter, subfamily G, member 2 (ABCG2) is crucial, and its reduced functioning leads to extra-renal underexcretion, resulting in a compensatory increase in renal urate output [8–11]. Therefore, it is essential to understand the functional and regulatory mechanisms of urate transport that could result in the development of new medications to control urate levels, especially for patients with chronic renal failure.

PDZ domain-containing 1 (PDZK1) is a scaffold protein that binds to several uric acid transporters and mediates their subcellular localization [12–14]. A single-nucleotide polymorphism, rs12129861, in the *PDZK1* gene is associated with serum uric acid [15, 16]. Shimizu et al. [17] reported that the expression of ABCG2 in the intestinal brush-border membranes was reduced in *Pdzk1*-knockout mice, suggesting that PDZK1 is significantly associated with the apical localization of ABCG2.

The clinical features of gout occur as a result of the inflammatory response to MSU crystals [2]. Accumulating evidence has demonstrated that MSU crystal-induced inflammation is a paradigm of innate immunity in gout [18]. Moreover, this inflammatory response is initiated when MSU crystals engaged the caspase-1-activating NOD-like receptor superfamily pyrin domain-containing 3 (NLRP3) inflammasome, resulting in the production of active interleukin-1β and interleukin-18. [19]. However, hyperuricemia is necessary but not sufficient for gout. Recent findings suggest that the presence of elevated soluble serum uric acid may also exert proinflammatory effects, even in the absence of gout [20–23]. Soluble uric acid increases NLRP3 inflammasome activation in human primary renal proximal tubule epithelial cells [24–26]. However, little is known concerning the effects and molecular mechanisms of uric acid in the intestines.

The aim of this article is to explore the relationship between soluble uric acid and gut excretion as well as the relevant mechanisms. In this study, HT-29 and Caco-2 cells were used as well-established models of human intestinal epithelial cells to examine the human intestinal transport mechanism. The results indicate that soluble uric acid increased the expression of PDZK1 and ABCG2 via the TLR4-NLRP3 inflammasome and phosphatidylinositol-4, 5-bisphosphate 3/kinase (PI3K)/protein kinase B (Akt) signaling pathway in HT-29 and Caco-2 cells. Stimulation of soluble uric acid also facilitated the translocation of ABCG2 from the intracellular compartment to the plasma membrane and increased its transport activity. An additional study, which was carried out to examine the possible interaction between *PDZK1* and *ABCG2*, indicated that *PDZK1* plays a pivotal role in the regulation of *ABCG2*.

## Methods

### Reagents and antibodies

Uric acid, lipopolysaccharide (LPS; from *Escherichia coli* 0111:B4), Brilliant Blue G, pyrrolidinedithiocarbamate (PTDC), and HEPES were purchased from Sigma-Aldrich (St. Louis, MO, USA). Wortmannin was purchased from MedChemExpress (Monmouth Junction, NJ, USA). Acetyl-YVAD-chloromethylketone and TAK242 were purchased from Calbiochem (Rockland, MA, USA). Pam3CSK4 was purchased from Tocris (Bristol, UK). Antibodies against phosphorylated-Akt (p-Akt), Akt, caspase-1 P10, and caspase-1 P20 were obtained from Cell Signaling Technology (Beverly, MA, USA). Antibodies against ABCG2, PDZK1, Na/K ATPase, Lamin A/C, GAPDH, β-actin, TLR2, TLR4, MYD88, P2X7, ASC, and nuclear factor-κB (NF-κB) were obtained from Santa Cruz Biotechnology (Santa Cruz, CA, USA). Penicillin/streptomycin and TRIzol reagent were purchased from Invitrogen Life Technologies (Carlsbad, CA, USA).

### Cell culture

HT-29 and Caco-2 human intestinal cell lines were purchased from the Cell Bank of the Chinese Academy of Sciences (Shanghai, China) and cultured in RPMI 1640 and high-glucose Dulbecco’s modified Eagle’s medium (DMEM) (Invitrogen) containing 10% fetal bovine serum (FBS; Gibco, Adelaide, Australia). Cells were grown in a humidified incubator containing 5% CO_2_ at 37 °C.

During the experiments, a growth arrest period in serum-free medium was observed overnight prior to stimulation. Cells were then treated with uric acid or the solvent (10 mM NaOH) after the addition of HEPES at a final concentration of 25 mM. The solution was filtered through a 0.22-μm pore size filter (Millipore, Shanghai, China) before use.

### Cellular stimulation conditions

The inhibitors were dissolved in DMSO or dd H_2_O. Cells were pretreated with the corresponding inhibitors in a humidified incubator containing 5% CO_2_ at 37 °C before stimulation with soluble uric acid. The final concentrations and incubation times were as follows: Brilliant Blue G (50 nM, 6 h), PTDC (100 μM, 2 h), Wortmanning (3 μg/ml, 2 h), acetyl-YVAD-chloromethylketone (20 μM, 2 h), TAK242 (2 μM, 2 h), Pam3CSK4 (5 μg/ml, 2 h), and LPS (1 μg/ml, 6 h).

### Extraction of subcellular fractions

For total protein extraction, cells were washed with ice-cold phosphate-buffered saline (PBS) and lysed in radioimmunoprecipitation assay lysis buffer supplemented with a proteasome inhibitor (Beyotime, Shanghai, China).

Nuclear and cytoplasmic extractions were prepared using an NE-PER Nuclear Cytoplasmic Extraction Reagent Kit (Pierce, Rockford, IL, USA) according to the manufacturer's instructions. Briefly, cells were washed by suspending the pellet in PBS. Next, ice-cold CER I was added to the cell pellet and vortexed vigorously on the highest setting for 15 s. The tube was then incubated on ice for 10 min. Ice-cold CER II was then added to the tube and vortexed for 5 s on the highest setting. The tube was incubated on ice for 1 min and vortexed again. The tube was centrifuged for 5 min at 16,000 × *g*, and the supernatant (cytoplasmic extract) was immediately transferred to a prechilled tube.

For cell membrane extraction, the Membrane Protein Extraction Kit (BioVision, Inc., Milpitas, CA, USA) was used according to the manufacturer’s instructions. In brief, cells were washed with ice-cold PBS and resuspended in Homogenization Buffer Mix in an ice-cold Dounce homogenizer. Cells were homogenized on ice 30–50 times and centrifuged at 700 × *g* for 10 min at 4 °C. The supernatant was collected and the pellet discarded. Cells were then centrifuged at 10,000 × *g* for 30 min at 4 °C. The pellet represents the cellular membrane protein, whereas the supernatant represents the cytosolic fraction. Membrane proteins were dissolved in 1 M urea.

### Western blot analysis

Equal amounts of protein were separated by 8–12% sodium dodecyl sulfate polyacrylamide gel electrophoresis and transferred to a polyvinylidene fluoride membrane (Millipore). The membrane was blocked in 5% nonfat dry milk for 2 h at room temperature and incubated overnight at 4 °C with the appropriate primary antibody: GAPDH (1:1000), ABCG2 (1:100), PDZK1 (1:500), MYD88 (1:1000), TLR2 (1:1000), TLR4 (1:1000), ASC (1:1000), NLRP3 (1:2000), caspase-1 P20 (1:1000), caspase-1 P10 (1:2000), P2X7 (1:1000), p-Akt (1:1000), Akt (1:1000), β-actin (1:1000), NF-κB p65 (1:1000), Na/K ATPase (1:1000), or Lamin A/C (1:1000). Horseradish peroxidase-conjugated goat anti-rabbit or goat anti-mouse IgG (1:5000; Cell Signaling Technology) was applied as a secondary antibody for 1 h at room temperature. Membranes were covered with enhanced chemiluminescence solution (Millipore) and exposed to film. Signal intensity was measured using a Bio-Rad XRS chemiluminescence detection system (Bio-Rad, Hercules, CA, USA).

### Immunofluorescence

HT-29 and Caco-2 cells were seeded onto 24-well plates. After treatment, cells were fixed in 4% paraformaldehyde for 15 min, washed with PBS, and permeabilized with or without 0.1% Triton X-100 (Beyotime) for 30 min. After blocking in 10% goat serum for 60 min, slides were incubated with a rabbit ABCG2 antibody (1:40) or a PDZK1 antibody (1:100) overnight at 4 °C. Samples were then incubated with Alexa Fluor 594-conjugated goat anti-mouse IgG antibody (Invitrogen) for 2 h, and nuclei were stained with 4′,6-diamidino-2-phenylindole (DAPI; Sigma-Aldrich). Samples were observed under a fluorescence microscope (Leica, Solms, Germany).

### Real-time quantitative polymerase chain reaction

Total RNA was isolated using TRIzol reagent (Invitrogen) and quantified by measuring the absorbance at 260 nm (NanoDrop 2000; Thermo Fisher Scientific, Waltham, MA, USA). Complementary single-stranded DNA was synthesized from total RNA by reverse transcription (PrimerScript^®^ RT Master Mix; TaKaRa, Kyoto, Japan). Each real-time PCR was performed in a total volume of 20 μl in duplicate using the SYBR^®^ Premix Ex Taq™ Kit (TaKaRa) on an ABI StepOnePlus System (Applied Biosystems, Warrington, UK). The following specific primers were used for amplification: GAPDH (forward 5′-AACTCCCACTCTTCCACCTTCG-3′ and reverse 5′-TCCACCACCCTGTTGCTGTAG-3′), PDZK1 (forward 5′-CAGCCTCACATTCTTCTT-3′ and reverse 5′-GGTCACAACTCATTCCTT-3′), and ABCG2 (forward 5′-AATACATCAGCGGATACTA-3′ and reverse 5′-AATAAGCCACCATCATAAG-3′). The cycle conditions were as follows: 95 °C for 30 s followed by 40 cycles at 95 °C for 5 s and 60 °C for 30 s. Relative gene expression was analyzed using the 2^−ΔΔCt^ method.

### Flow cytometry

A detailed protocol for the MDR assay is available in the e-Fluxx-ID^®^ Green Multidrug Resistance Assay Kit (ENZO Life Sciences, Inc., Farmingdale, NY, USA) instruction manual. Briefly, on the day of the assay, cells were collected, washed with PBS, and incubated with or without the ABCG2 inhibitor novobiocin in the presence of e-Fluxx-ID^®^ Green for 30 min at 37 °C. Propidium iodide was added to cells during the last 5 min of incubation and analyzed immediately on a flow cytometer (FACSAriaSORP; BD Diagnostics, Franklin Lakes, NJ, USA) equipped with a blue (488 nm) laser, and the signals were registered in the FL1/FITC (530/30 filter) channel. Data analysis was performed using FlowJo 8.8.2 software.

To analyze ABCG2 activity, the multidrug resistance factor (MAF) value was calculated using the following formula for each probe:

$$ \mathrm{MAF}=100\times \left(\mathrm{MFI}\;\mathrm{of}\kern0.17em \mathrm{novobiocin}\hbox{-} \mathrm{treated}\kern0.17em \mathrm{cells}\hbox{--} \mathrm{MFI}\;\mathrm{of}\kern0.17em \mathrm{untreated}\kern0.17em \mathrm{cells}\right)/\mathrm{MFI}\;\mathrm{of}\kern0.17em \mathrm{novobiocin}\hbox{-} \mathrm{treated}\kern0.17em \mathrm{cells}, $$

where MFI = mean fluorescence intensity.

### Transfection of human intestinal cells with small interfering RNA

For small interfering RNA (siRNA) transfection, cells were plated onto six-well plates and cultured in RPMI 1640 or DMEM without FBS and antibiotics overnight before siRNA knockdown. siRNA transfections were carried out using Lipofectamine^®^ 2000 (Invitrogen) according to the manufacturer's instructions. Briefly, 10 μl siRNA and 5 μl Lipofectamine^®^ 2000 reagent were combined in a total of 300 μl Opti-MEM I (Gibco, Invitrogen). Thereafter, 700 μl Opti-MEM I was added to the mixture, and the mixture was added to each well. After incubation for 6 h, fresh DMEM or RPMI 1640 containing 5% FBS was added to each well. Cells were returned to the incubator for an additional 48–72 h. The negative control siRNA (scrambled-siRNA) (GenePharma, Shanghai, China) was used to account for nonsequence-specific effects. The siRNA sequences are as follows: ABCG2 siRNA sense:5′-GGAGGCAAAUCUUCGUUAUTT-3′ and antisense 5′-AUAACGAAGAUUUGCCUCCTT-3′; and PDZK1 siRNA sense 5′-GAUGGAGACAGAGUUCUUATT-3′ and antisense: 5′-UAAGAACUCUGUCUCCAUCTT-3′.

### Statistical analysis

Statistical analysis was performed using GraphPad Prism 5.0 software (San Diego, CA, USA). All experiments were performed at least in triplicate, and the data are presented as the mean ± standard error of the mean (SEM). Statistical significance was determined using one-way analysis of variance followed by Tukey’s multiple comparison test when comparing more than two groups. *P* ≤ 0.05 was considered to represent a statistically significant difference.

## Results

### Expression of PDZK1 and ABCG2 in human intestinal cells is mediated by stimulation of soluble uric acid

**Fig. 1:**
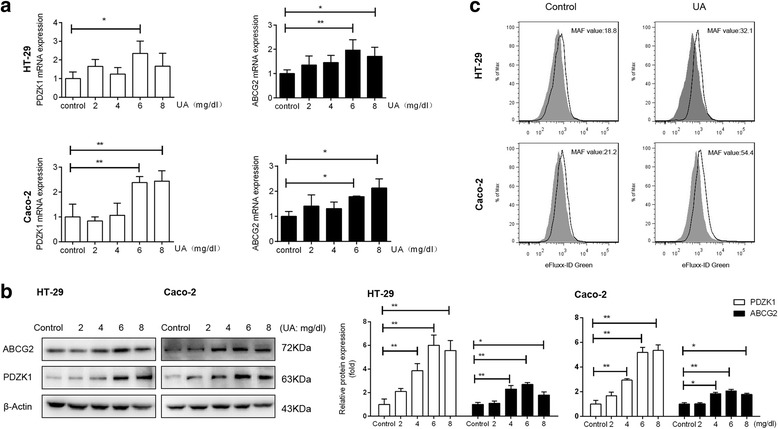
PDZK1 and ABCG2 expression mediated by stimulation with soluble urate in HT-29 and Caco-2 cells. Cells treated with soluble urate or 10 mM NaOH for 24 h. **a** Relative mRNA levels of PDZK1 and ABCG2 determined by RT-qPCR. Data presented as mean ± standard error of the mean (SEM). *P < 0.05 and **P < 0.01, compared to control cells; *n* = 3. **b** Representative western blots of PDZK1 and ABCG2. Protein expression normalized to that of GAPDH. **c** Efflux function of ABCG2 evaluated by detecting the intracellular fluorescence of e-Fluxx-ID^®^ Green Dye with or without the ABCG2 inhibitor, novobiocin. Tinted histograms show fluorescence of untreated cells, and nontinted histograms show fluorescence of inhibitor-treated cells. Multidrug resistance activity factor (MAF) value is indicative of corresponding protein activity. ABCG2 ATP-binding cassette transporter, subfamily G, member 2, PDZK1 PDZ domain containing 1, UA uric acid

Human intestinal cells were exposed to various concentrations of soluble uric acid (2, 4, 6, or 8 mg/dl) or 10 mM NaOH for 24 h. The mRNA expression of PDZK1 and ABCG2 increased dramatically after treatment with 6 and 8 mg/dl soluble uric acid. Real-time quantitative polymerase chain reaction (RT-qPCR) analysis revealed the increases in PDZK1 and ABCG2 mRNA expression respectively in both cell lines compared to control cells (Fig. 1a). Meanwhile, PDZK1 and ABCG2 expression was significantly increased in soluble uric acid-treated cells compared to control cells (Fig. 1b). Cells were also treated with 6 mg/dl soluble uric acid for 2, 6, 12, 24, 36, or 48 h. The expression of PDZK1 and ABCG2 peaked at 24 h (*P* < 0.01, compared to control cells) (see Additional file 1). Accordingly, this effect was prominent at the concentration of 6 mg/dl for 24 h in both HT-29 and Caco-2 cells, and this was used for subsequent experiments.

The function of ABCG2 in HT-29 and Caco-2 cells was examined with e-Fluxx-ID^®^ Green Dye with and without a specific inhibitor. Tinted histograms revealed a difference in fluorescence between inhibitor-treated and untreated samples, indicative of ABCG2 protein activity (according to the MAF values). The MAF values revealed weak inhibition on ABCG2 efflux function in both HT-29 and Caco-2 wild-type cells. However, after stimulation with soluble uric acid, the MAF value (32.1) increased significantly, by 70.7%, in HT-29 cells. In Caco-2 cell lines, ABCG2 activity was more than two-fold higher compared to that in wild-type cells (Fig. 1c).

### Soluble uric acid altered the subcellular localization of PDZK1 and ABCG2 in human intestinal cells

**Fig. 2:**
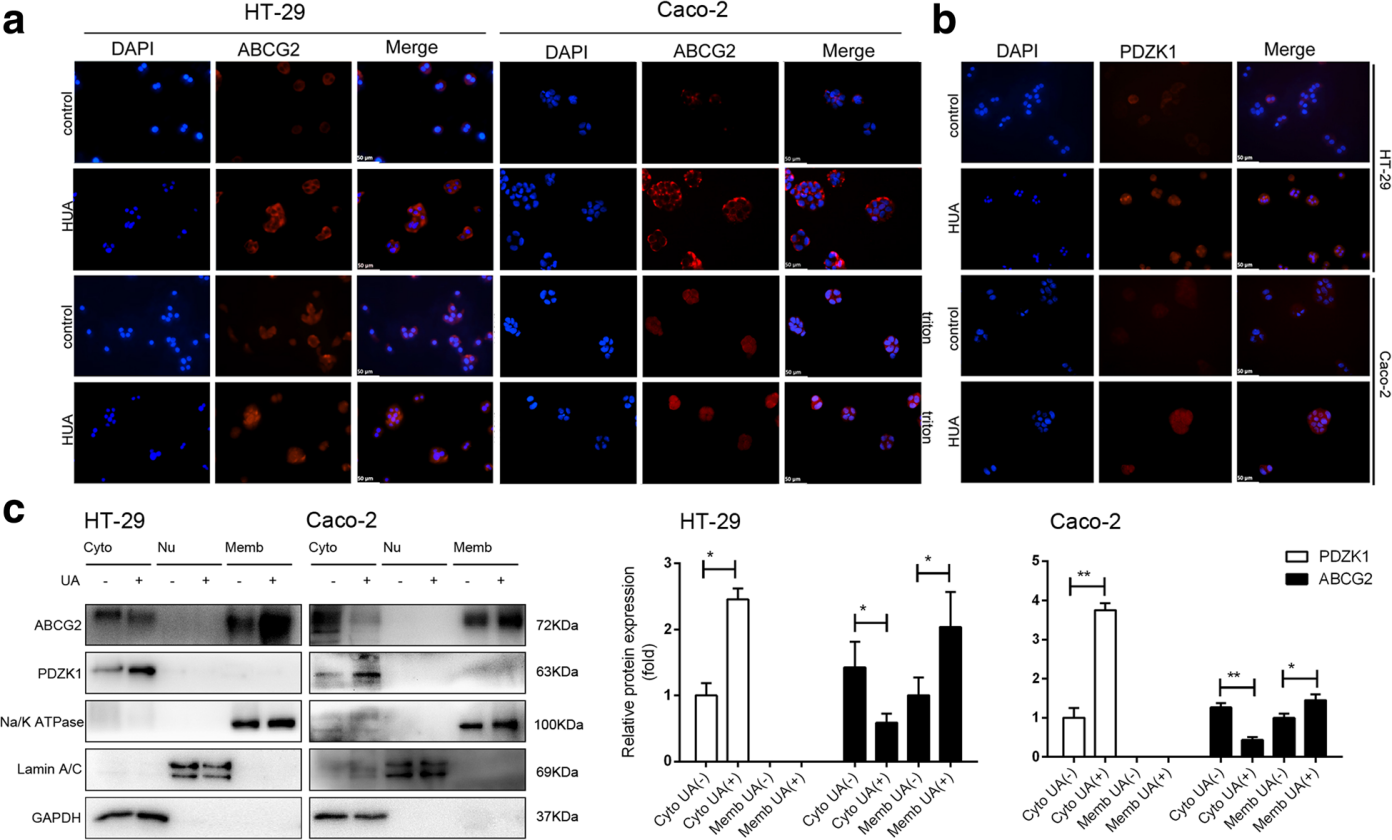
Soluble urate altered subcellular localization of PDZK1 and ABCG2 in HT-29 and Caco-2 cells. Cells treated with 6 mg/dl soluble urate or 10 mM NaOH for 24 h. **a** Cells fixed with paraformaldehyde and permeabilized with or without Triton X-100 and stained with BXP21 anti-ABCG2 antibody (red). Nuclei stained with DAPI (blue). Scale bar = 50 μm. **b** Immunofluorescence staining using an antibody against PDZK1 (red). Nuclei stained with DAPI (blue). Scale bar = 50 μm. **c** Subcellular distribution of PDZK1 and ABCG2 in HT-29 and Caco-2 cells. Cytoplasmic (Cyto, lanes 1 and 2), nuclear (Nu, lanes 3 and 4), and membranous (Memb, lanes 5 and 6) extracts prepared from cells and used for western blot analyses. Data are presented as the mean ± SEM. *P<0.05 and **P<0.01, compared to control cells; *n* = 3. GAPDH used as a cytoplasmic fraction marker; Lamin A/C used as a nuclear marker; and Na/K ATPase used as a membrane marker. Cytoplasmic fraction normalized to that of GAPDH, whereas membrane fraction normalized to that of Na/K ATPase. ABCG2 ATP-binding cassette transporter, subfamily G, member 2, DAPI 4′,6-diamidino-2-phenylindole, GAPDH glyceraldehyde-3-phosphate dehydrogenase, PDZK1 PDZ domain containing 1, UA uric acid, HUA high concentrations of uric acid (8mg/dl)

To further understand the subcellular distribution of PDZK1 and ABCG2 proteins, we performed immunofluorescence on HT-29 and Caco-2 cells. The ABCG2 signals increased significantly in the membrane after treatment with soluble uric acid. Nevertheless, there were no obvious changes when cells were treated with Triton X-100 (Fig. 2a), probably because ABCG2 protein was present not only in the membrane fractions but also in the cytoplasm. Subcellular fractionation methods were also used to quantify the protein amount in each subcellular compartment. The cytoplasmic fraction was marked by the presence of GAPDH, whereas the membrane fraction contained Na/K ATPase, and the nuclear fraction was enriched in nuclear lamina protein Lamin A/C. Western blot analysis demonstrated that soluble uric acid upregulated the expression of ABCG2 in the membrane fraction and downregulated its expression in the cytoplasm (Fig. 2c). Furthermore, consistent with the immunofluorescence results, ABCG2 protein was not observed in the nucleus (Fig. 2a, c). Together, these findings suggest that soluble uric acid induces the membrane translocation of ABCG2 in HT-29 and Caco-2 cells.

Immunofluorescence staining and cell fractionation studies revealed that PDZK1 protein was present in the cytoplasm and exhibited increased expression upon stimulation with soluble uric acid (Fig. 2b, c).

### PDZK1 regulated the expression and function of ABCG2 in human intestinal cells

**Fig. 3:**
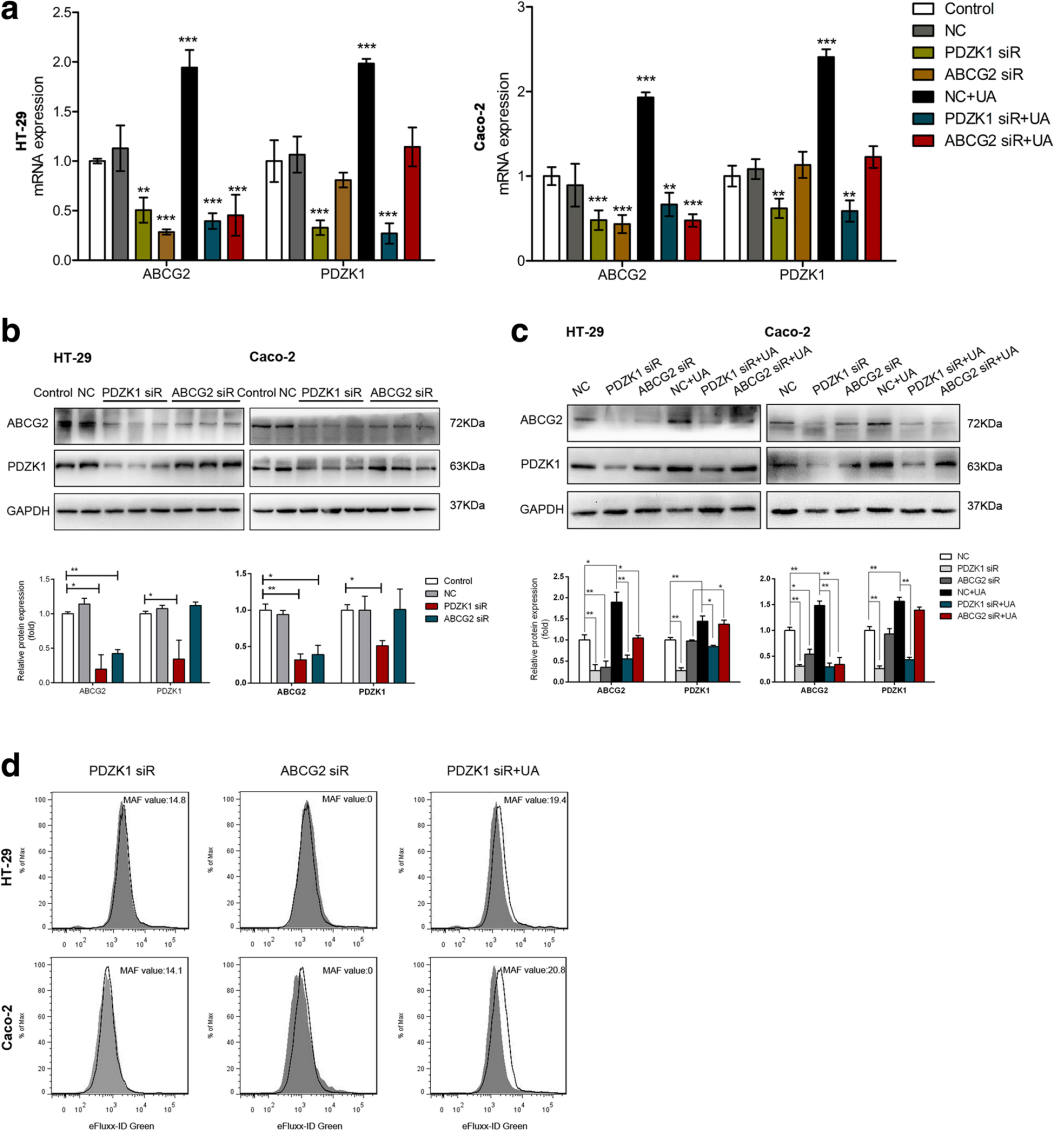
PDZK1 regulated expression and function of ABCG2 in human intestinal cells. Cells transfected with ABCG2 or PDZK1 siRNA or scrambled siRNA for 48 h. Cells then incubated with 6 mg/dl soluble urate or 10 mM NaOH for 24 h. **a** Relative mRNA levels of PDZK1 and ABCG2 determined by RT-qPCR. Data presented as mean ± SEM. **P < 0.01 and ***P < 0.001, compared to control cells; *n* = 3. **b c** Representative western blot analysis of PDZK1 and ABCG2. Protein expression normalized to that of GAPDH. Data are presented as the mean ± SEM. *P<0.05 and **P<0.01, compared to control cells; *n* = 3. **d** Efflux function of ABCG2 evaluated by detecting the intracellular fluorescence of e-Fluxx-ID^®^ Green Dye with or without the ABCG2 inhibitor, novobiocin. Tinted histograms show fluorescence of untreated cells, and nontinted histograms show fluorescence of inhibitor-treated cells. MAF value is indicative of corresponding protein activity. ABCG2 ATP-binding cassette transporter, subfamily G, member 2, GAPDH glyceraldehyde-3-phosphate dehydrogenase, PDZK1 PDZ domain containing 1, UA uric acid, NC negative control, siR small interfering RNA

The results reported indicate the simultaneous regulation of PDZK1 and ABCG2, suggesting their interaction. To address this hypothesis, siRNAs were used to knock down PDZK1 and ABCG2 in HT-29 and Caco-2 cells. Negative control cells were transfected with a scrambled siRNA. PDZK1 and ABCG2 siRNAs strongly attenuated the expression of their corresponding transcripts, as determined by western blot and RT-qPCR analyses; the decreases in expression were statistically significant (*P* < 0.01, compared to the controls) (Fig. 3a, b). It is also important to note that PDZK1 siRNA reduced ABCG2 expression at both the mRNA and protein levels. In HT-29 and Caco-2 cells, the mRNA expression of ABCG2 was reduced by approximately 50% after PDZK1 siRNA transfection (*P* < 0.01, compared to the control) (Fig. 3a). After transient transfection of ABCG2 siRNA, the mean fluorescence intensity value corresponding to inhibitor-treated cells was lower than that of untreated cells (Fig. 3d). In such cases, the corresponding MAF values would be regarded as zero according to the manufacturer’s instructions. ABCG2 activity was suppressed by PDZK1 siRNA compared to negative control cells. As shown in Figs. 1c and 3d, the MAF values decreased to 21% and 33% in HT-29 and Caco-2 cells, respectively.

Moreover, soluble uric acid failed to increase the expression of ABCG2 after PDZK1 knockdown. The decreases in ABCG2 mRNA expression were 40% and 66% in HT-29 and Caco-2 cells respectively. No significant differences were observed in PDZK1-knockdown cells (*P* > 0.05, compared to control cells) (Fig. 3a). A significant decrease in ABCG2 protein was observed after PDZK1 siRNA transfection, regardless of the presence of uric acid (Fig. 3b, c). The MAF values of 19.4 and 20.8 increased slightly after treatment with soluble uric acid in PDZK1-knockdown cells. Compared to the uric acid-treated group, the MAF values decreased to 39% and 62% respectively (Figs. 1c and 3d). On the other hand, knockdown of ABCG2 did not affect PDZK1 expression (Fig. 3a, b). Soluble uric acid increased the expression of PDZK1 protein after transient transfection of ABCG2 siRNA relative to soluble uric acid-treated cells (Fig. 3c). The mRNA level did not differ significantly from that of the control group (*P* > 0.05), but was lower than that of the soluble uric acid-treated group (*P* < 0.01) (Fig. 3a).

### Soluble uric acid upregulated expression of PDZK1 and ABCG2 by activating the TLR4/NLRP3/caspase-1 inflammasome

**Fig. 4:**
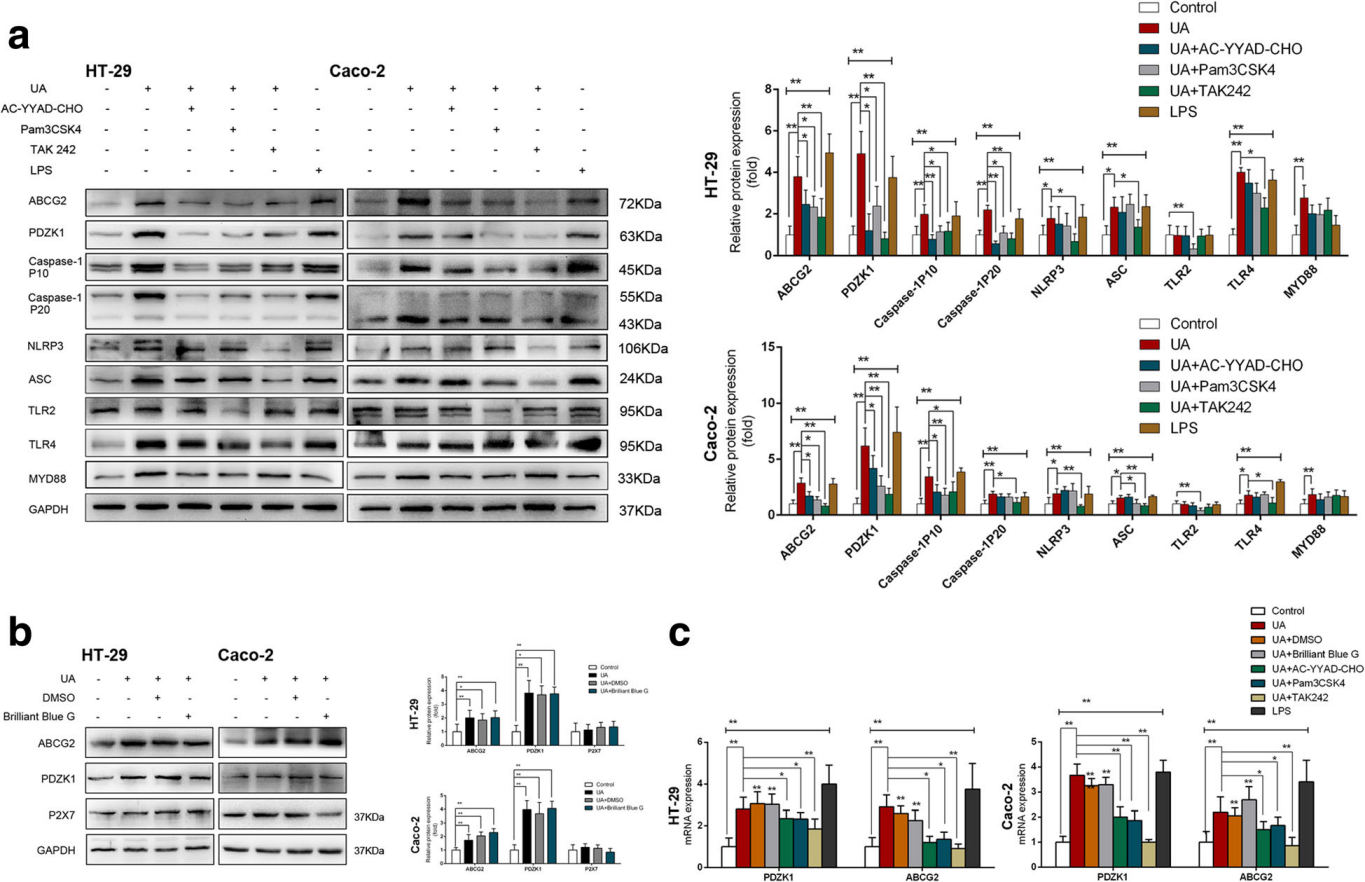
Soluble urate activated the TLR4-NLRP3 inflammasome, and increased expression of PDZK1 and ABCG2 was regulated by the TLR4-NLRP3 inflammasome. Cells preincubated with the caspase-1 inhibitor Ac-YVAD-CMK (20 μM), the TLR1/2 ligand Pam3CSK4 (5 μg/ml), the TLR4 inhibitor TAK-242 (2 μM), or DMSO-Control for 2 h or with P2X7 inhibitor Brilliant Blue G (50 nM) for 6 h. Cells then incubated with 6 mg/dl soluble urate or 10 mM NaOH for 24 h in continued presence or absence of the inhibitor. Indicated cells incubated with 1 μg/ml lipopolysaccharide (LPS) for 6 h. **a b** ABCG2, PDZK1, pro-caspase-1, active caspase-1 (p10), NLRP3, TLR2, TLR4, MYD88, and P2X7 measured by western blot analysis. Protein expression normalized to that of GAPDH. **c** Relative mRNA levels of PDZK1 and ABCG2 determined by RT-qPCR. Data presented as mean ± SEM. *P < 0.05 and **P < 0.01, compared to control cells; *n* = 3. Ac-YVAD-CMK acetyl-YVAD-chloromethylketone, ABCG2 ATP-binding cassette transporter, subfamily G, member 2, ASC apoptosis-associated speck-like protein-containing a CRAD, DMSO dimethylsulfoxide, GAPDH glyceraldehyde-3-phosphate dehydrogenase, LPS lipopolysaccharide, NLRP3 NOD-like receptor superfamily, pyrin domain containing 3, PDZK1 PDZ domain containing 1, TLR Toll-like receptor, UA uric acid

To investigate the molecular mechanism of the upregulation of PDZK1 and ABCG2 by soluble uric acid, expression of the TLR4/NLRP3/caspase-1 inflammasome was assessed. Soluble uric acid upregulated expression of the NLRP3 inflammasome, which is comprised of NLRP3, apoptosis-associated speck-like protein-containing a CRAD (ASC), and caspase-1, indicating that soluble uric acid leads to the production of active caspase-1 (Fig. 4a). We also investigated the proteins located upstream of caspase-1 in the inflammatory response. Western blot analysis showed no effect on the expression of Toll-like receptor 2 (TLR2) or P2X7, but increases in TLR4 and myeloid differentiation primary response 88 (MYD88) were observed after treatment with soluble uric acid (Fig. 4a, b).

Next, cells were pretreated with the corresponding inhibitors before stimulation. PDZK1 and ABCG2 expression was quantified by RT-qPCR and western blot analyses. Treatment with the caspase-1 inhibitor acetyl-YVAD-chloromethylketone (Ac-YVAD-CMK, 20 μM) inhibited the increased expression of PDZK1 and ABCG2 induced by soluble uric acid (Fig. 4a, c), whereas the P2X7 inhibitor Brilliant Blue G (50 nM) did not (Fig. 4b, c). Furthermore, the TLR1/2 ligand Pam3CSK4 (5 μg/ml) inhibited the expression of caspase-1, as well as PDZK1 and ABCG2, indicating that active caspase-1 is relevant in the upregulation of PDZK1 and ABCG2 (Fig. 4a, c). Treatment with TAK-242 (2 μM), a small-molecule specific inhibitor of TLR4 signaling, suppressed the soluble uric acid-induced expression of NLRP3, ASC, and caspase-1. Inhibitory effects of PDZK1 and ABCG2 were also observed (Fig. 4a, c). To further explore whether the soluble uric acid-induced increases in PDZK1 and ABCG2 were involved in the TLR4/NLRP3/caspase-1 inflammasome, cells were treated with LPS (1 μg/ml), a potent inducer of the inflammatory response mediated by TLR4. The expression of PDZK1 and ABCG2, as well as of the proteins involved in the TLR4/NLRP3/caspase-1 inflammasome, increased dramatically compared to the control (Fig. 4a, c), suggesting a key role for TLR4.

### The soluble uric acid-induced increases in PDZK1 and ABCG2 are partially dependent on PI3K/Akt signaling

**Fig. 5:**
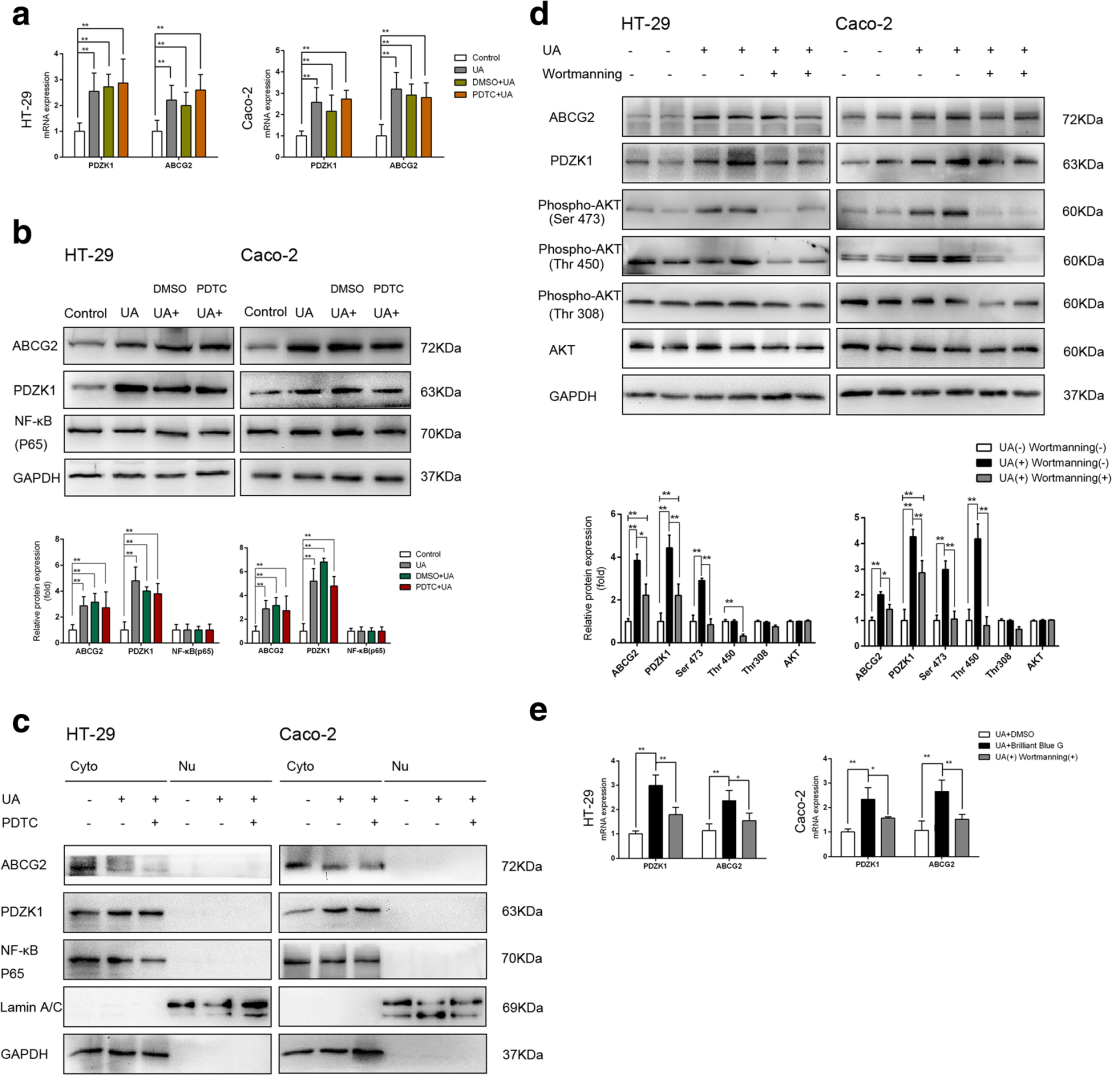
Soluble urate-induced increases in PDZK1 and ABCG2 expression partially dependent on PI3K/Akt signaling. Cells preincubated with or without the NF-κB inhibitor PDTC (100 μM), the PI3K inhibitor Wortmannin (3 μg/ml), or DMSO-Control for 2 h. Cells then incubated with 6 mg/dl soluble urate or 10 mM NaOH for 24 h. **a** Relative mRNA levels of PDZK1 and ABCG2 determined by RT-qPCR. Data presented as mean ± SEM. *P < 0.05 and **P < 0.01, compared to control cells; *n* = 3. **b d** Total protein expression of ABCG2, PDZK1, Akt, p-Akt, and NF-κB p65 determined by western blot analysis. Protein expression normalized to that of GAPDH. **c** Subcellular distribution of NF-κB p65, PDZK1, and ABCG2 in HT-29 and Caco-2 cells. Cytoplasmic (Cyto, lanes 1, 2, and 3) and nuclear (Nu, lanes 4, 5, and 6) extracts prepared from cells and used for western blot analyses. GAPDH used as a cytoplasmic fraction marker; Lamin A/C used as a nuclear marker. ABCG2 ATP-binding cassette transporter, subfamily G, member 2, DMSO dimethylsulfoxide, GAPDH glyceraldehyde-3-phosphate dehydrogenase, NF-κB nuclear factor-kappa B, PDZK1 PDZ domain containing 1, PDTC pyrrolidinedithiocarbamate, UA uric acid

Next, we explored whether the soluble uric acid-induced increases in PDZK1 and ABCG2 were associated with the NF-κB or PI3K/Akt signaling pathway. As treatment with soluble uric acid did not modulate the total protein of NF-κB p65 (Fig. 5a, b), we used subcellular fractionation to investigate whether p65 translocated from the cytoplasm to the nucleus. Soluble uric acid did not activate the NF-κB signaling pathway by classic nuclear translocation of the p65 subunit (Fig. 5c). Meanwhile, the soluble uric acid-induced increases in PDZK1 and ABCG2 expression were not suppressed by the NF-κB inhibitor, pyrrolidinedithiocarbamate (PTDC, 100 μM) (Fig. 5a, b).

Soluble uric acid activated Akt by phosphorylating Ser473 and Thr450 on in Caco-2 cells and Ser473 in HT-29 cells (Fig. 5d). Moreover, this phosphorylation of Akt was determined to be PI3K-dependent via inhibition with Wortmannin (2 μg/ml). Conversely, inhibition of PI3K by Wortmannin partially reduced the increased expression of PDZK1 and ABCG2 at both the mRNA and protein levels (Fig. 5d, e), suggesting that PI3K/Akt signaling may participate in the soluble uric acid-induced effects.

## Discussion

**Fig. 6:**
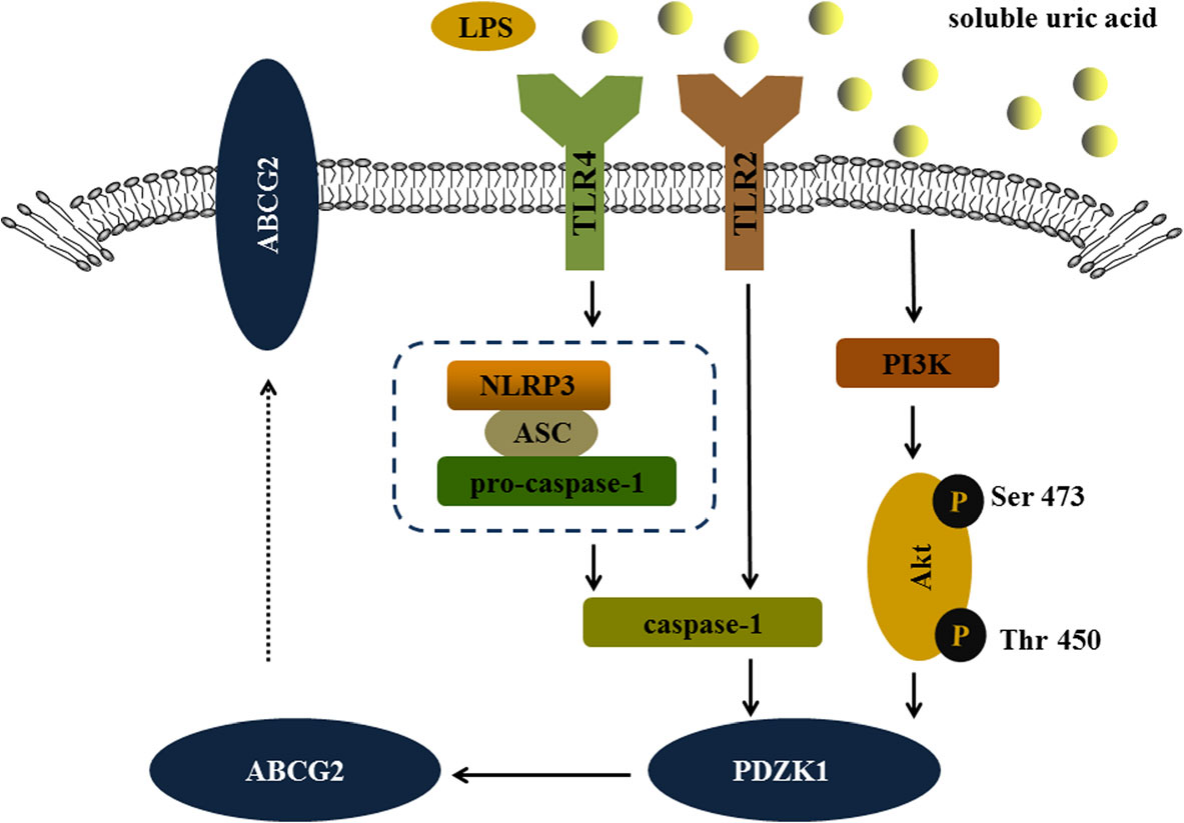
TLR4-NLRP3 inflammasome and PI3K/Akt signaling pathway modulated expression of PDZK1 and ABCG2 stimulated by soluble uric acid in human intestinal cells. Soluble uric acid interacted with TLR2 or TLR4, initiating the formation of caspase-1 by recruiting the NLRP3 inflammasome, which is comprised of NLRP3, ASC, and pro-caspase-1, and activated the PI3K/Akt signaling pathway by phosphorylating Ser473 and Thr450 on Akt. Activation of caspase-1 and Akt increased the expression of PDZK1, which upregulated the expression of ABCG2. ABCG2 ATP-binding cassette transporter, subfamily G, member 2, ASC apoptosis-associated speck-like protein-containing a CRAD, LPS lipopolysaccharide, NLRP3 NOD-like receptor superfamily, pyrin domain containing 3, PDZK1 PDZ domain containing 1, PI3K/Akt phosphatidylinositol-4, 5-bisphosphate 3-kinase/protein kinase B, TLR Toll-like receptor, UA uric acid

A previous study demonstrated that soluble uric acid may act as a proinflammatory agent, independent of its precipitated form in MSU crystals [23], providing mechanistic insight into the immunomodulatory properties of soluble uric acid that could be attributed to feedback regulation of urate transporters. Although researchers are becoming increasingly aware that decreased extra-renal urate excretion caused by ABCG2 dysfunction is a common mechanism of hyperuricemia [8, 11, 27], the effect of soluble uric acid on urate excretion is not completely understood. In this study, we show a relevant link between soluble uric acid and the gut excretion (Fig. 6). And for the first time, we show a mechanism for the upregulation and transport activities of gut urate excretion (Fig. 6).

HT-29 and Caco-2 cells, which are well-established models of human intestinal epithelial cells, were used to examine the human intestinal transport mechanism. ABCG2, a high-capacity urate exporter, facilitates uric acid secretion in the intestine rather than the proximal tubule of the kidney [8]. These experiments revealed that soluble uric acid could also attribute to a feedback regulation of the urate transporters in human intestinal cell lines.

Stimulation of soluble uric acid appears to facilitate the translocation of ABCG2 from the intracellular compartment to the plasma membrane, where it mediates renal urate secretion. A more robust transport assay (i.e., using membrane vesicles from HEK293 cells expressing ABCG2) verified its role as a high-capacity urate transporter [28]. Cycling of membrane transporters between the plasma membrane and intracellular sites may serve as a general regulatory paradigm for the expression and activity of transporters at the cell surface [29]. Utilizing this mechanism, the plasma membrane permeability for certain substances can be rapidly changed to allow cells to respond to varying physiologic conditions [29].

PDZK1 binds to several urate transporters at its PDZ domain, and well-characterized PDZ domain-containing proteins regulate the trafficking and activity of multiple transport proteins in the proximal tubule [13]. In addition to ABCG2, these transport proteins include urate transporter 1 (URAT1/SLC22A12) [30], organic anion transporter 10 (OAT10/SLC22A13) [31], organic anion transporter 4 (OAT4/SLC22A11) [32], and others [15]. Shimizu et al. [17] reported that PDZK1 is a functional regulator and directly interacts with ABCG2 at the protein level. However, there is no evidence that the expression of ABCG2 is affected by the genetic depletion of PDZK1. The present study suggests that this regulation occurs at the transcriptional level in human intestinal cells. Moreover, soluble uric acid failed to increase the expression and function of ABCG2 after knockdown of PDZK1, indicating that increased expression of ABCG2 mediated by uric acid is dependent on PDZK1.

Thus far, major studies investigating the proinflammatory effects of uric acid have focused on MSU crystal-induced inflammation and immunity-related components [17, 22]. Since uric acid was first considered a signal sensed by innate immunity (including TLR4 activation and NLRP3 inflammasome in gouty arthritis) [25, 26, 33], the role of uric acid metabolism in immune activation and inflammation has become the current direction and hotspot concerning gout. Previous studies examining the role of soluble uric acid have mainly focused on the kidney (both renal tubules [34] and glomerular cells [26]). In the current study, we determined that soluble uric acid activates the TLR4-dependent NLRP3 inflammasome in human intestinal cells. Moreover, the NLRP3 inflammasome plays an important role in the regulation of PDZK1 and ABCG2. However, previous studies have demonstrated decreased expression of PDZK1 in both a mouse model of chronic colitis [35] and in humans with inflammatory bowel disease [36], also suggesting that the regulation of inflammatory mediators is the probable mechanism of PDZK1 expression.

Although increasing evidence has demonstrated that TLRs and MyD88-dependent NF-κB signaling pathways are both involved in MSU crystal-mediated gouty arthritis [19, 37], soluble uric acid was revealed to have no effect on the NF-κB signaling pathway in this study.

Canonical TLR signaling by parallel pathways also involves PI3K/Akt in chondrocytes stimulated with MSU and calcium pyrophosphate dehydrate crystals [19, 38]. Akt, a serine/threonine kinase, is critical for the mediation of cell signaling initiated by growth factors, cytokines, and other cellular stimuli [39]. Inhibition of the PI3K/Akt signaling pathway was recently shown to modulate ABCG2-mediated drug transport via the translocation of ABCG2 from the plasma membrane to intracellular compartments in different cell systems, including side population cells in the bone marrow [40], glioma-derived stem-like cells [41], and ABCG2-overexpressing extracellular vesicles derived from MCF-7 breast cancer cells [42]. Our findings showed that soluble uric acid activated PI3K/Akt signaling via the phosphorylation of Ser473 and Thr450 on Akt, and its effects on PDZK1 and ABCG2 were modified by the PI3K inhibitor, Wortmannin.

There were several limitations to this study. First, it is possible that the TLR4/NLRP3 inflammasome and the PI3K/Akt signaling pathway interact in response to soluble uric acid. Second, it should be noted that any other mechanisms involved in the inflammatory effects of soluble uric acid cannot be excluded, and this merits further investigation. More importantly, although demonstrated in vitro, these findings should be confirmed in primary intestine cells, as well as in animal models for translational relevance.

## Conclusions

This study set out to investigate the functional and regulatory mechanisms of gut excretion. We show a relevant link between soluble uric acid and the gut excretion. We also show a mechanism for the upregulation of PDZK1 and ABCG2 via the TLR4-NLRP3 inflammasome and PI3K/Akt signaling pathway. Moreover, PDZK1 plays a pivotal role in the regulation of ABCG2. This research will serve as a base for future studies and provide insights for understanding mechanisms of hyperuricemia.

## Additional files

Additional file 1: Figure S1. Human intestinal cells exposed for various times. Cells treated with 6 mg/dl soluble uric acid for 2, 6, 12, 24, 36, or 48 h. (**A**) Relative mRNA levels of PDZK1 and ABCG2 determined by RT-qPCR. Data presented as mean ± standard error of the mean (SEM). *P < 0.05 and **P < 0.01, compared to control cells; *n* = 3. (**B**) Representative western blot assays of PDZK1 and ABCG2 (JPG 435 kb)

## Abbreviations

ABCG2: ATP-binding cassette transporter, subfamily G, member 2
ASC: Apoptosis associated speck like protein containing a CRAD
BSA: Bovine serum albumin
DAPI: 4′,6-Diamidino-2-phenylindole
DMEM: Dulbecco’s modified Eagle’s medium
DMSO: Dimethyl sulfoxide
EDTA: Ethylenediaminetetraacetic acid disodium salt
ELISA: Enzyme-linked immunosorbent assay
FBS: Fetal bovine serum
GAPDH: Glyceraldehyde-3-phosphate dehydrogenase
LPS: Lipopolysaccharide
MSU: Monosodium urate
MYD88: Myeloid differentiation factor 8
NF-κB: Nuclear factor-kappa B
NLRP3: NOD-like receptor superfamily, pyrin domain containing 3
P2X7: Purinergic receptor P2X ligand-gated ion channel 7
PBS: Phosphate buffer saline
PCR: Polymerase chain reaction
PDTC: Pyrrolidinedithiocarbamate
PDZK1: PDZ domain containing 1
PI3K/Akt: Phosphatidylinositol-4, 5-bisphosphate 3-kinase/protein kinase B
siRNA: small interfering RNA
TLR: Toll-like receptor
