# Supplementary figures and images for: Soluble uric acid increases PDZK1 and ABCG2 expression in human intestinal cell lines via the TLR4-NLRP3 inflammasome and PI3K/Akt signaling pathway
Source: Arthritis Res Ther. 2018 Feb 7;20:20. doi: 10.1186/s13075-018-1512-4 (PMC5803867; doi:10.1186/s13075-018-1512-4)

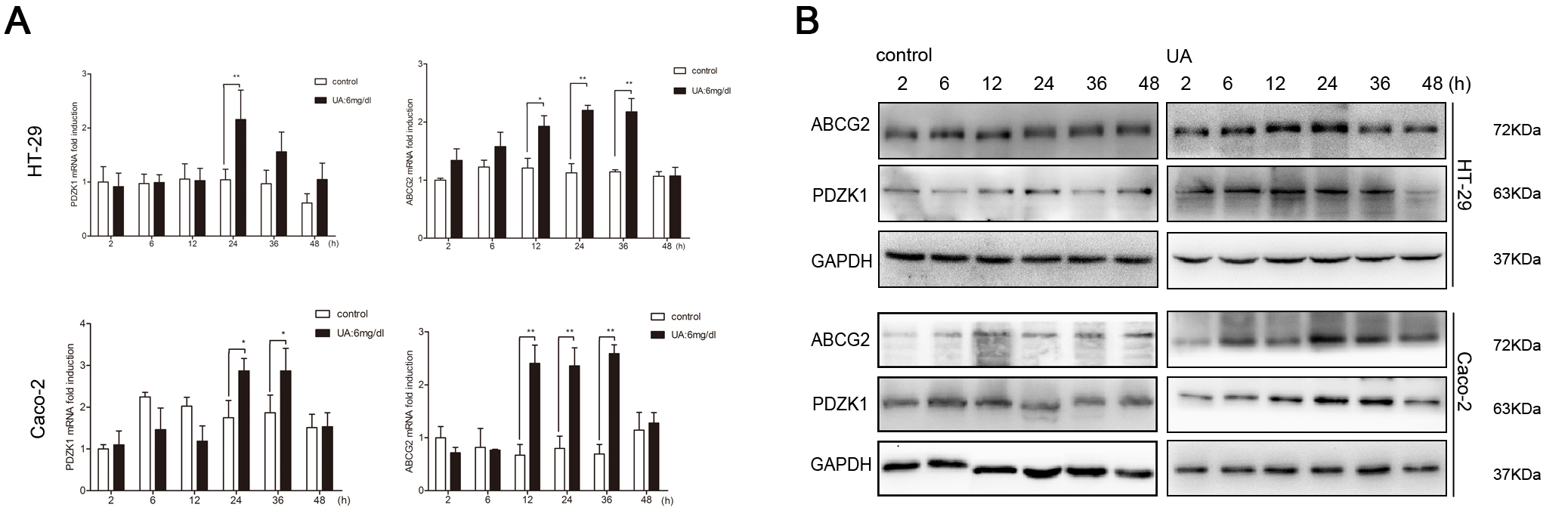

Supplement: Supplementary file 1 — Human intestinal cells exposed for various times. Cells treated with 6 mg/dl soluble uric acid for 2, 6, 12, 24, 36, or 48 h. (A) Relative mRNA levels of PDZK1 and ABCG2 determined by RT-qPCR. Data presented as mean ± standard error of the mean (SEM). *P < 0.05 and **P < 0.01, compared to control cells; n = 3. (B) Representative western blot assays of PDZK1 and ABCG2 (JPG 435 kb) [file 13075_2018_1512_MOESM1_ESM.jpg]
